# Phosphorylation of ADP-Glucose Pyrophosphorylase During Wheat Seeds Development

**DOI:** 10.3389/fpls.2020.01058

**Published:** 2020-07-10

**Authors:** Danisa M. L. Ferrero, Claudia V. Piattoni, Matías D. Asencion Diez, Bruno E. Rojas, Matías D. Hartman, Miguel A. Ballicora, Alberto A. Iglesias

**Affiliations:** ^1^ Laboratorio de Enzimología Molecular, Instituto de Agrobiotecnología del Litoral (UNL-CONICET) & FBCB, Santa Fe, Argentina; ^2^ Department of Chemistry and Biochemistry, Loyola University Chicago, Chicago, IL, United States

**Keywords:** crop grasses, glucan accumulation, starch biosynthesis, post-translational modification, enzyme regulation

## Abstract

Starch is the dominant reserve polysaccharide accumulated in the seed of grasses (like wheat). It is the most common carbohydrate in the human diet and a material applied to the bioplastics and biofuels industry. Hence, the complete understanding of starch metabolism is critical to design rational strategies to improve its allocation in plant reserve tissues. ADP-glucose pyrophosphorylase (ADP-Glc PPase) catalyzes the key (regulated) step in the synthetic starch pathway. The enzyme comprises a small (S) and a large (L) subunit forming an S_2_L_2_ heterotetramer, which is allosterically regulated by orthophosphate, fructose-6P, and 3P-glycerate. ADP-Glc PPase was found in a phosphorylated state in extracts from wheat seeds. The amount of the phosphorylated protein increased along with the development of the seed and correlated with relative increases of the enzyme activity and starch content. Conversely, this post-translational modification was absent in seeds from *Ricinus communis*. *In vitro*, the recombinant ADP-Glc PPase from wheat endosperm was phosphorylated by wheat seed extracts as well as by recombinant Ca^2+^-dependent plant protein kinases. Further analysis showed that the preferential phosphorylation takes place on the L subunit. Results suggest that the ADP-Glc PPase is a phosphorylation target in seeds from grasses but not from oleaginous plants. Accompanying seed maturation and starch accumulation, a combined regulation of ADP-Glc PPase by metabolites and phosphorylation may provide an enzyme with stable levels of activity. Such concerted modulation would drive carbon skeletons to the synthesis of starch for its long-term storage, which later support seed germination.

## Introduction

Starch is a major product of photosynthesis performed by vascular plants and constitutes the foremost storage of carbon and energy in grasses (Jeon et al., 2010; Pandey et al., 2012; Tuncel and Okita, 2013; MacNeill et al., 2017; Goren et al., 2018). Chemically, starch is a mix of two homopolysaccharides, amylose and amylopectin; which are mainly composed of glucose units linked by α-1,4-bonds. Amylose is essentially a linear α-1,4-glucan with scarce branches of α-1,6-bonds, while amylopectin contains a high number of α-1,6-linked branch points (Pandey et al., 2012; MacNeill et al., 2017; Goren et al., 2018). Cereals (wheat, maize, barley, and rice as more relevant in production) store starch in seed endosperm, and they supply more than half of the caloric demands in the world population (Tuncel and Okita, 2013; Goren et al., 2018). Enhanced biosynthesis of the polysaccharide greatly influences the grain yield of cereals having starch as the principal reserve compound (Jeon et al., 2010; Tuncel and Okita, 2013; MacNeill et al., 2017; Goren et al., 2018; Zi et al., 2018). Improvement (in quantity and quality) in the production of key harvestable grains is a challenge to be solved in the coming decades. Indeed, it is projected a critical demographic expansion together with increasing industrial requirements of feedstock for biofuels, bioplastics, and bioadhesives to cope with climate change by the mid-century (Morell and Myers, 2005; Godfray et al., 2010; Tuncel and Okita, 2013; Iglesias, 2015; Goren et al., 2018). In this scenario, the in-depth understanding of the process of starch biosynthesis is relevant to better design strategies to increase yields in plants of agronomic interest.

Starch accumulates in plastids of both photosynthetic and non-photosynthetic plant cells (Jeon et al., 2010; MacNeill et al., 2017; Goren et al., 2018). The linear α-1,4-glucan is elongated by the starch synthases, which use ADP-glucose (ADP-Glc) as the glycosyl donor molecule. Synthesis of this sugar nucleotide takes place from glucose-1-phosphate (Glc1P) and ATP in a reaction catalyzed by ADP-Glc pyrophosphorylase (EC 2.7.7.27, ADP-Glc PPase) (Ballicora et al., 2003; Ballicora et al., 2004). The metabolic route follows by the action of enzymes implicated in branching, debranching, phosphorylation, and de-phosphorylation of the polymer under formation (Wilkens et al., 2018). Most of these enzymes arise in isoforms exhibiting changes in specificity and ability to interact with different partners forming multi-protein complexes of functional relevance for the production of the polysaccharide (Crofts et al., 2017; Wilkens et al., 2018). The step producing ADP-Glc is rate-limiting in the starch biosynthetic pathway (Kavakli et al., 2001b; Ballicora et al., 2003; Ballicora et al., 2004; Tuncel and Okita, 2013). ADP-Glc PPase is present in bacteria (where it is involved in glycogen synthesis) and green plants. The enzyme from different sources is allosterically regulated by metabolites that are critical intermediates of the central carbon energy metabolism operating in the respective organism (Ballicora et al., 2003; Ballicora et al., 2004). ADP-Glc PPase from cyanobacteria (Iglesias et al., 1991), green algae (Iglesias et al., 1994), and higher plants (Ballicora et al., 2004) have 3P-glycerate (3PGA) and inorganic orthophosphate (Pi) as the principal allosteric activator and inhibitor, respectively; with some enzyme promiscuity toward being activated by hexose-Ps (Kuhn et al., 2013).

The ADP-Glc PPase presents in green algae and higher plants is a heterotetramer (S_2_L_2_), composed of small (S, 50-53 kDa) and large (L, 54-60 kDa) subunits (Ballicora et al., 2003; Ballicora et al., 2004). Subunits S and L are homologous proteins, where the L polypeptide has emerged in different species *via* gene duplication followed by subfunctionalization (Ballicora et al., 2005; Georgelis et al., 2008; Georgelis et al., 2009; Kuhn et al., 2009; Ferrero et al., 2018; Figueroa et al., 2018). In potato tuber, the S subunit retained the catalytic function, whereas the L subunit specialized in modulating the regulation of the former (Ballicora et al., 2005). Even so, different alternatives exist where the L subunit is also catalytic, or it presents isoforms within the same organism (Crevillén et al., 2003; Ventriglia et al., 2008; Kuhn et al., 2009). The interaction between both subunits is determinant for the enzyme activity and regulatory responses (Kavakli et al., 2001a; Baris et al., 2009). As demonstrated in previous studies (Crevillén et al., 2003; Ferrero et al., 2018), the L subunit confers the sensitivity to allosteric regulators exhibited by heterotetrameric ADP-Glc PPases from Arabidopsis and wheat. Excluding the enzyme from monocot (as wheat) endosperm, the ADP-Glc PPase S subunit from leaves and other plant tissues has an N-terminal cysteine residue that is critical for redox regulation. This latter involves the formation of a disulfide bridge between the S subunits in the heterotetramer, mediated by the thioredoxin system (Ballicora et al., 1999; Ballicora et al., 2000; Tiessen et al., 2002). In addition, as it has been reviewed previously (Ballicora et al., 2004), ADP-Glc PPases found in cereal endosperm have distinct regulatory properties. In the barley and maize forms, 3PGA modifies the relative affinity for substrates (Plaxton and Preiss, 1987; Kleczkowski et al., 1993). Also, in wheat (*Triticum aestivum)* endosperm enzyme, neither 3PGA nor fructose-6P (Fru6P) modify the *V*_max_, but they increase the relative affinity for Glc1P by 2-fold (Gómez-Casati and Iglesias, 2002; Ferrero et al., 2018). On the other hand, the 3PGA and Fru6P revert the inhibition by Pi (Gómez-Casati and Iglesias, 2002).

A substantial body of experimental evidence is giving support to the modulation of starch biosynthesis by the combined action of post-translational mechanisms, including redox modification, protein complex formation, and protein phosphorylation (Lohrig et al., 2009; Kötting et al., 2010; Geigenberger, 2011; Ma et al., 2014; Tetlow et al., 2015; Goren et al., 2018). In this context, proteomic information obtained in the last decade draws attention to ADP-Glc PPase as a putative target of protein kinases (Lohrig et al., 2009; Kötting et al., 2010; Ma et al., 2014). Specifically, a proteomic analysis of maize endosperm identified the phosphorylation of the small subunit of the enzyme (Yu et al., 2019). However, all these predictive results lack functional and developmental evidence of the tangible presence of the enzyme at a phosphorylated state *in planta*. The latter is critical for the complete understanding of factors affecting plant productivity, thus limiting the design of better strategies for its improvement. Herein, we report the *in vivo* phosphorylation of ADP-Glc PPase associated with the development of wheat seeds. This modification was further analyzed by *in vitro* studies working with recombinant enzymes, specifically wheat endosperm ADP-Glc PPase, as well as Ca^2+^-dependent SOS2 and CDPK plant protein kinases. Results suggest that phosphorylation of the enzyme involved in the limiting step of starch built-up would be functionally relevant for the yield of grains in grass crops.

## Materials And Methods

### Chemicals

ATP, Fru6P, 3PGA, and Glc1P were from Sigma Aldrich (St. Louis, MO, USA). All other reagents were of the highest quality available.

### Seeds Harvest

Seeds harvesting was as described previously (Piattoni et al., 2017). Briefly, *Triticum aestivum* L. cv. Baguette 11 samples collected at 3, 6, 10, 14, 17, and 27 days post-anthesis (DPA), and spikes were frozen immediately in liquid nitrogen. This sampling was based on reports indicating that the complete growth of wheat seeds is reached at 45 DPA, establishing the following respective phases of development: cell proliferation 0–10 DPA, accumulation or reserves 11–30 DPA, and seed maturation and desiccation 30–45 DPA (http://bio-gromit.bio.bris.ac.uk/cerealgenomics/WheatBP/Documents/DOC_WheatBP.php). Wheat samples were grains taken from the central part of the frozen spike (between the fifth and tenth spike) stored at -80°C until analysis. Castor (*Ricinus communis*) seeds were collected at 5, 10, 20, 25, 32, and 40 days post-pollination (DPP). The sampling criterion considered that castor oil seed development completes in ~60 days (Canvin, 1963), after which six groups classified according to the morphology exhaustively described in (Greenwood and Bewley, 1982). Sampled seeds were dissected from the capsule, frozen immediately in liquid nitrogen, and store at -80°C until analysis.

### Soluble Protein Extraction From Seeds

Seeds whole protein extraction was made by triplicate (using independent biological replicates) as reported elsewhere (Piattoni et al., 2017). Frozen seeds of wheat or castor oil were ground to a fine powder in liquid nitrogen using a mortar and pestle. It followed the addition of 1 µl cold fresh prepared extraction buffer per 1 mg of frozen powdered tissue. Composition of the extraction buffer was 50 mM MOPS pH 8.0, 1 mM EDTA, 1 mM EGTA, 25 mM NaF, 0,1% (v/v) Triton X-100, 20% (v/v) glycerol, 10 mM MgCl_2_, 2 mM DTT, 4% (p/v) PEG-8000, 2 mM PMSF, 5 mM malic acid, and 1% (p/v) polyvinylpyrrolidone (Turner et al., 2005). This buffer was supplemented with 2 mM aminocaproic acid, 1 mM benzamidine, 10 mM NaF, 1 mM Na_2_MoO_4_, 1 mM Na_2_VO_4_, and SETIII (1X) protease inhibitor cocktail EDTA-Free (Calbiochem). The mixture was incubated 20 min on ice with constant homogenization. Extracts were centrifuged 30 min at 4°C and 15,000 × g. The supernatant was recovered and used immediately for the different assays.

### Starch and Lipid Quantification

Quantification of starch from seeds was as indicated in (Piattoni et al., 2017), following a protocol that combines two previously described procedures (Reibach and Benedict, 1982; Baud and Graham, 2006). Plant tissue (100 mg) ground in a mortar under liquid nitrogen was soaked with 500 ml of ethanol 95% (v/v) at 4°C and then centrifuged 10 min at 15,000 × g and 4°C. The supernatant was discarded and the extraction repeated thrice to eliminate soluble sugars. The pellets were dried at 60°C, weighed, and then dissolved in 10 ml of distilled H_2_O per mg of extracted material. The resulting tubes (hermetically closed) boiled for 1 h to solubilize the starch, and then centrifuged during 10 min at 15,000 ×g and 4°C. The soluble fraction (20 µl) was added to 200 µl of 100 mM sodium acetate pH 4.5 plus 70 U of amyloglucosidase (1,4-α-D-glucan glucohydrolase) and incubated 16 h at 55–60°C for starch digestion. After centrifuging for 10 min at 15,000 × g and 4°C, the resulting soluble sugars were quantified by an enzymatic colorimetric assay where the H_2_O_2_ produced by glucose oxidase is measured by a peroxidase coupled to a colorimetric compound. The reaction mixture (100 µl) consisted of 70 µl of the commercial reactive (10 kU/l glucose oxidase, 1 kU/l peroxidase, 0.5 mM 4-aminophenazone, 100 mM phosphate buffer pH 7.0, and 12 mM 4-hydroxybenzoates) and 30 µl of sample conveniently diluted. The reaction was developed for 10 min at 37°C and the product quantified at 492 nm. To correlate the quantity of starch and the concentration of soluble sugars, we constructed a calibration curve [glucose (mg/ml) versus starch (mg)] with a standard starch solution treated identically to the sample.

To determine the contents of TAGs in seeds, we utilized protocols already described (Folch et al., 1957; Piattoni et al., 2017). Plant tissue (200 mg) was ground to a fine powder in liquid nitrogen and lipids extracted with 0.2 ml of MilliQ H_2_O and 3.8 ml of chloroform/methanol: 2/1 (v/v) solution. The extraction was performed in hermetically closed tubes incubated 2 h at room temperature with gentle mixing every 30 min. Samples were filtered on oil-free filter paper previously washed with the extraction solution, placed in previously weighed tubes, and vigorously mixed with 0.7 ml of 0.02% CaCl_2_ solution in chloroform/methanol/H_2_O: 3/48/47 (v/v). After centrifugation at 3,000 × g for 5 min and discard the upper phase, 0.7 ml of chloroform/methanol/H_2_O: 3/48/47 (v/v) were added, following vigorous mixing. Samples were centrifuged as before until phase separation, and the upper phase was discarded by suction, then evaporating chloroform at 45–50°C to obtain the lipids. After weighing, the lipids dissolved in isopropanol served to quantify TAGs by an enzymatic colorimetric assay. This method was based on the treatment of TAGs with lipase, then converting the produced glycerol into glycerol-3P by a specific kinase coupled to the reaction of glycerol-3P oxidase that produces H_2_O_2_. The latter was quantified by employing a peroxidase generating a compound that absorbs at 492 nm. The reaction mixture contained 50 mM PIPES pH 7.5, 5 mM 4-chlorophenol, 15 kU/l lipase, 1 kU/l glycerol-3P kinase, 2.5 U/ml glycerol-3P oxidase, 0.44 kU/l peroxidase, 0.7 mM 4-aminophenasone, 0.18 mM ATP, and the lipid sample.

All values of starch and lipids contents are the mean of at least three independent determinations, and reproducible within ±10%.

### Enzyme Activity Assay

ADP-Glc PPase activity was determined at 37°C in ADP-Glc synthesis direction by following Pi formation (after hydrolysis of PP_i_ by inorganic pyrophosphatase) using the highly sensitive colorimetric method previously described (Fusari et al., 2006). The reaction mixture contained 100 mM MOPS pH 8.0, 7 mM MgCl_2_, 1.5 mM ATP, 1.0 mM Glc1P, 0.2 mg/ml BSA, 0.5 U/ml yeast inorganic pyrophosphatase and a proper sample enzyme dilution. Assays initiated by the addition of Glc1P at a final concentration of 1.5 mM in a total volume of 50 µl. The reaction mixture was incubated for 10 min at 37°C and terminated by adding the Malachite Green reactive. The complex formed with the released P*_i_* was measured at 630 nm. One unit of activity (U) is the amount of enzyme catalyzing the formation of 1 µmol of ADP-Glc per minute under the described conditions. All determinations are the mean of at least three independent sets of data that were reproducible within ±10%.

### Protein Methods

Total protein quantification was determined by the method of Bradford (Bradford, 1976) using BSA as a standard. Protein electrophoresis in polyacrylamide gels under denatured conditions (SDS-PAGE) was performed as previously described by Laemmli (Laemmli, 1970). Western blotting was performed by transferring the proteins resolved by SDS-PAGE to nitrocellulose membranes using a Mini-PROTEAN II (Bio-Rad) apparatus. The membrane was blocked 2 h at room temperature and subsequently incubated with primary antibody during 16 h at 15°C with agitation. The primary antibodies were raised in rabbits against the ADP-Glc PPase purified from spinach leaves (Gómez-Casati and Iglesias, 2002) or against each subunit of the wheat endosperm enzyme (*Tae*S and *Tae*L) produced in our laboratory. After intensive washing, membranes were incubated with HRP−conjugated anti−rabbit secondary antibody (Sigma) for 1 h. Bands were visualized using the ECL method and detection reagents (Thermo Scientific).

For the phosphorylated protein mobility delay assay, Phos-tag™ (Wako Chemicals) was added (100 µM) to the 10% (w/v) SDS-PAGE acrylamide gel. The acrylamide-pendant Phos-tag ligand provides a molecule with a functional affinity to interact with phosphate groups, after which produce electrophoretic mobility-shifts of phosphorylated proteins (Kinoshita et al., 2015). The phosphorylation reaction was carried out as described below except for the use of non-radioactive ATP and modifying the reaction time to 2 h with the addition of the recombinant kinase (and the corresponding amount of buffer according to the final volume change) every 30 min. Then, samples were denatured with SDS-PAGE buffer [1% SDS (w/v), 100 mM β-mercaptoethanol, in 50 mM TRIS-HCl pH 6.8] at 100°C for 5 min. After the electrophoretic run (30 mA per gel), gels were washed twice in methanol-free Tris-Gly transfer buffer and 1 mM EDTA, for 10 min each time with gentle agitation, followed other 10 min in methanol-free transfer buffer without EDTA. It followed electrotransference of the proteins and immunodetection with specific antibodies.

### Phosphorylated Proteins Purification and De-Phosphorylation

Purification of phosphorylated proteins was performed by Fe^3+^-Immobilized metal affinity chromatography (IMAC-Fe^3+^), as previously described (Muszyńska et al., 1986). Total proteins (1.2 mg) in 50 mM MES-NaOH pH 6.0 were loaded onto 100 ml of iminodiacetic acid-Fe^3+^ previously equilibrated with the same buffer. After 1 h incubation at room temperature and constant homogenization, non-adsorbed proteins were washed out twice with 2 ml of 50 mM MES-NaOH, pH 6.0. The increase of pH in three steps was then employed to elute the adsorbed. First, 5 volumes of 50 mM PIPES-HCl pH 7.2 were applied, then the adsorbed proteins were washed out with three volumes of 50 mM Tris-HCl pH 8.0, and finally phosphorylated proteins (tightly bound to the matrix) eluted with two volumes of 50 mM of Tris-HCl pH 9.0. SDS-PAGE served to resolve the samples eluted at the different pH conditions and for the analysis of the phosphorylated proteins.

The protein purification was performed from three independent biological replicates to enable an assessment of significance.

Protein de-phosphorylation experiments followed a protocol described elsewhere (Bustos and Iglesias, 2002). Proteins (600 µg) extracted from seeds were diluted in 50 mM Tris–HCl (pH 8.5), 1 mM EDTA, 10 mM MgCl_2_, 1.2 mM CaCl_2_, 20 mM 2-mercaptoethanol, 1mM PMSF, and 20 U of alkaline phosphatase (Promega). Samples were incubated at 37°C for 5 h, and the reaction stopped by the addition of 4X SDS-PAGE sample buffer. Then, samples were analyzed by electrophoresis followed by western blotting and immunodetection.

### ADP-Glc PPase and Protein Kinases Cloning, Expression, and Purification

The genes coding for the small (*Tae*S) and large (*Tae*L) subunits of wheat endosperm ADP-Glc PPase were synthesized *de novo* from DNA sequence reported previously (Ainsworth et al., 1993 and Ainsworth et al., 1995) and subcloned into pET28c as reported elsewhere (Ferrero et al., 2018). This recombinant enzyme displayed similar properties to the ADP-Glc PPase purified from the wheat endosperm (Gómez-Casati and Iglesias, 2002). For individual expression, each subunit was subcloned into the pET19TEV vector using *Nde*I and *Xho*I. For coexpression, *Tae*S was subcloned from the pET28c plasmid into the pCDFDuet-1 vector using *Nde*I and *Xho*I sites. By combining the pCDFDuet-1/*Tae*S and pET19TEV/*Tae*L constructs, we obtained the heterotetrameric wheat endosperm ADP-Glc PPase (*Tae*SL) with a His-tag only in the L subunit. All the sequences were confirmed by the University of Chicago DNA Sequencing Facility (Chicago, IL, United States).

The *Ath*SnRK1α1 gene amplified from *A. thaliana* cDNA served to generate the S198D mutation [required for its activity, as reported by (Shen et al., 2009)], by employing the QuickChange Site-Directed Mutagenesis kit (Agilent) (Kunkel et al., 1991). The mutated gene was subcloned in the pETDuet-1 vector to produce a protein with an N-term His-tag. The *Mdo*SOS2 gene was synthesized *de novo* with a sequence and codon usage optimized for its expression in *E. coli*. The T168D mutation [required for activity (Guo, 2001)] was introduced by quick-change mutagenesis and then subcloned into a pET28b vector to produce a protein with an N-term His-tag. Molecular cloning of the gene coding for *Stu*CDPK1 was as already reported (Santin et al., 2017; Rojas et al., 2018).

Recombinant *Tae*S, *Tae*L and *Tae*SL were obtained from *E. coli* BL21(DE3) RIL cells transformed with [pET19TEV/*Tae*S], [pET19TEV/*Tae*L] and co-transformed with [pCDFDuet-1/*Tae*S + pET19/*Tae*L], respectively. Recombinant *Ath*SnRK1α1 was obtained from *E. coli* BL21 Shuffle cells transformed with [pETDuet-1/*Ath*SnRK1α1]; *Mdo*SOS2 was obtained from *E. coli* BL21 Codon Plus cells transformed with [pET28b/*Mdo*SOS2] and *Stu*CDPK1 was obtained from *E. coli* BL21 Shuffle cells transformed with [pET22^(+)^/*Stu*CDPK1]. Transformed cells were grown in 1 L of LB medium supplemented with the appropriate antibiotic (100 µg/ml ampicillin for pET19TEV, pET22^(+)^, and pETDuet-1; 50 µg/ml kanamycin for pET28b, and 100 µg/ml for pCDFDuet-1) at 37 °C and 200 rpm until OD600 reached ~1.2. Cells were induced with 0.5 mM isopropyl-β-D-1-thiogalactopyranoside (IPTG) at 25°C and 180 rpm for 16 h and then harvested by centrifuging 10 min at 4°C and 5000 x*g*. The cells were resuspended in Buffer H [50 mM Tris-HCl pH 8.0, 300 mM NaCl, 5% (v/v) glycerol] and disrupted by sonication on ice (4 s pulse on with intervals of 3 s pulse off for a total time of 5 min). After centrifuging twice at 30,000 x*g* for 10 min, the supernatant (crude extract) was loaded onto a 1 ml HisTrap column (GE Healthcare) previously equilibrated with Buffer H. The recombinant protein was eluted with a linear gradient from 10 to 300 mM imidazole in Buffer H, and fractions containing the highest activity (or more pure proteins) were pooled with 10% (v/v) glycerol and stored at -80°C until use. The pools of *Tae*SL, *Tae*L and *Tae*S, were dialyzed against Buffer X [50 mM MOPS pH 8.0, 0.1 mM EDTA, 20% (w/v) sucrose, 5 mM MgCl_2_] and concentrated using an Amicon Ultra-4 30 K unit (Millipore, Billerica, MA, USA). Under these conditions the enzyme was fast frozen and stored at -80°C until use, being fully actives for at least 1 year (in the case of *Tae*SL).

### *In Vitro* Phosphorylation Assays

For *in vitro* phosphorylation of recombinant forms of the wheat endosperm ADP-Glc PPase [the heterotetrameric (*Tae*SL), small subunit (*Tae*S), or large subunit (*Tae*L); see (Ferrero et al., 2018), the respective purified enzyme (2 µg) was incubated under conditions established for the activity of different protein kinases from plants (Piattoni et al., 2011; Rojas et al., 2018)]. The three (I–III) specific phosphorylation conditions used for assay crude extracts from seeds or purified recombinant protein kinase were as follows. I. For SnRK or Ca^2+^-independent kinases: 100 mM HEPES pH 7.3, 5 mM DTT, 10 mM MgCl_2_, 0.05 mM ATP, 0.5 mM EGTA. II. For CDPK or Ca^2+^-dependent kinases: 20 mM Tris-HCl pH 7.5, 10 mM MgCl_2_, 5 mM DTT, 1 mM CaCl_2_, 0.05 mM ATP. III. For SOS2: 20 mM Tris-HCl pH 7.2, 5 mM MgCl_2_, 5 mM DTT, 0.5 mM CaCl_2_, 0.01 mM ATP, 2.5 mM MgCl_2_. Worthy of mention is that the critical difference between these protein kinases is related to requirement (or not) of calcium. In contrast, all of them need reducing condition (5 mM DTT) for optimal activity (Raíces et al., 2001; Crozet et al., 2010; Gong et al., 2019). Each reaction (final volume 20 µl) was incubated at 30°C for 30 min after the addition of 1 µCi of [^32^P]-γ-ATP (Migliore-Lacaustra) and initiated by adding wheat endosperm extract as kinase resource, or an appropriate aliquot of the recombinant protein kinase [0.4 mU (determined for AMARA peptide)]. After the reaction, the protein mixtures were denatured with SDS-PAGE buffer [1% SDS (w/v), 100 mM β-mercaptoethanol, in 50 mM Tris-HCl pH 6.8]. It followed the resolution of the proteins by electrophoresis [SDS-PAGE with 10% (w/v) acrylamide and run at 30 mA/gel]. Then, gels were stained with Coomassie Brilliant Blue R-250, dried, and radioactivity incorporation detected by Storing Phospho-screen (GE Healthcare) exposure and scanning with the Typhoon™ system (GE Healthcare). All phosphorylation assays were performed from three independent technical replicates to enable an assessment of significance.

## Results and Discussion

### ADP-Glc PPase Is Phosphorylated Along With Development of the Seed in Wheat but Not in Castor Bean

**Figure 1:**
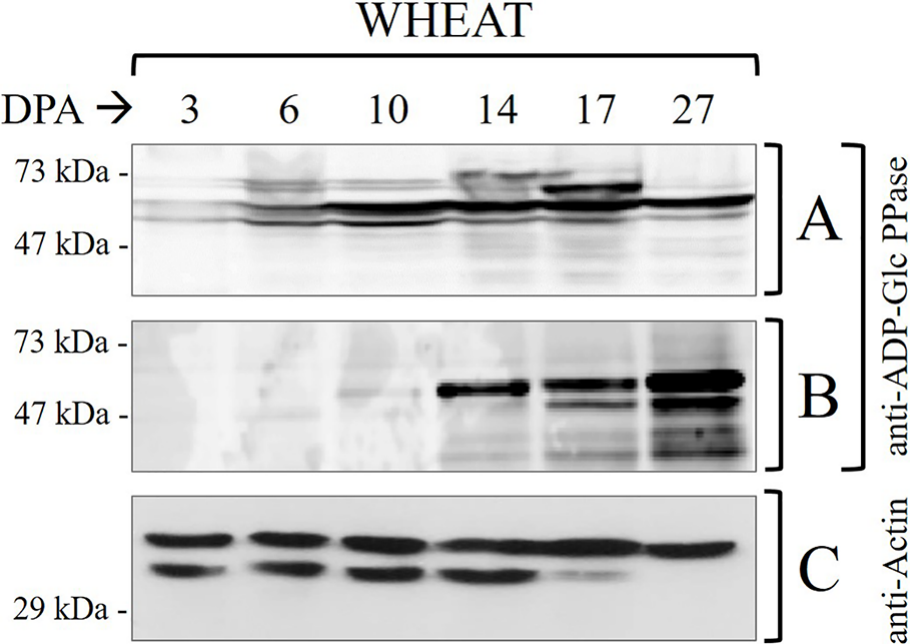
Immunodetections in wheat seed samples throughout development: **(A)** total proteins and **(B)** phosphoproteins purified by IMACFe3+ evaluated with anti-ADP-Glc PPase [obtained from the purified enzyme of leaves of *Spinacia oleracea* (Gómez-Casati and Iglesias, 2002)] **(C)** control of actin in total proteins. Crude extracts preparation and phosphoprotein purification were performed from three independent biological replicates. Protein profiles of the samples are shown in **Supplemental Figure 2**.

Seeds of grasses are relevant components within the world crops providing food and energy, with wheat being one of the principal edible grains. From this, it is clear the relevance of starch as a major staple. Another primary product of agriculture is TAGs accumulated in seeds of oleaginous plants. To better understand starch biosynthesis (and its regulation) in seeds, we explored about contents of the polysaccharide and TAGs, as well as ADP-Glc PPase activity and phosphorylation profiles along with the development of *Triticum aestivum* (wheat) seeds (**Table 1** and **Figure 1**). For comparison, we performed similar studies with seeds of the oleaginous *Ricinus communis* (castor bean) (**Supplemental Table 1** and **Supplemental Figure 1**). As shown in **Table 1**, wheat seeds stored significantly more starch than TAGs, with levels of the polysaccharide progressively increasing along the stages of development, reaching levels up to 30% of the seed weight during accumulation of reserves (between 11–30 DPA). These profiles are in agreement with values reported in previous studies on developing wheat endosperm (Briarty et al., 1979; Dai, 2010; Piattoni et al., 2017). Instead, castor bean seeds exhibited a progressive increase in the amount of starch during cell proliferation (0–20 DPP), but an abrupt decrease during accumulation of reserves (20–40 DPP) and maturation plus desiccation (30–50 DPP). As reported for the oleaginous plant (Canvin, 1963; Greenwood and Bewley, 1982), TAGs remained at a low level during cell proliferation switching to an increase during accumulation to reach up to 30% of the seed weight at 50 DPP (**Supplemental Table 1**).

**Table 1:** Determinations of weight, starch, and TAG contents, ADP-Glc PPase activity and total soluble proteins in wheat seeds at different development stages. DPA, days post-anthesis.

Determinations	WHEAT (DPA)					
	3	6	10	14	17	27
**Seed weight (mg/seed)**	1.39	1.07	2.67	6.94	11.23	22.61
**Starch (mg/seed)**	0.02	0.08	0.22	0.75	1.98	7.01
**TAG (mg/seed)**	1.10^-4^	1.10^-4^	0.001	0.008	0.004	0.107
**ADP-Glc PPase activity** **(U/mg)**	0.131	0.303	0.232	0.408	0.616	0.597
**Total soluble proteins (mg/seed)**	0.023	0.016	0.032	0.090	0.127	0.314
**Events**	*Cell* *proliferation*			*Transition*	*Storage/* *Maturation*	

The determinations are the mean of at least three independent set of data that were reproducible within ±10%.

It is worth correlating levels of starch and ADP-Glc PPase activity in **Table 1** with profiles of the enzyme immunodetected in extracts (either whole protein or phosphoprotein enriched after IMAC-Fe^3+^) from wheat seeds at the different development stages detailed in **Figure 1**. As expected from the critical role played by ADP-Glc PPase in starch biosynthesis, the specific activity determined for the enzyme followed a moderate increase between the cell proliferation, transition, and maturation steps of development of wheat grains, accompanying levels of the polysaccharide accumulation (**Table 1**). Thus, the enzyme activity increased ~2-fold between 3 and 10 DPA and other ~2.5-fold between 10 and 27 DPA. The profile of the enzyme immunodetection showed a continued increase in protein levels between 3 and 10 DPA, then remaining almost similar between 10 and 17 DPA with a slight decrease at 27 DPA (**Figure 1A**). It is also evident that since 14 DPA (with small increases at 17 and 27 DPA), ADP-Glc PPase was found as a phosphoprotein in wheat seed extracts (**Figure 1B**). Concerning castor bean seeds, values of the specific activity (**Supplemental Table 1**) and protein levels (**Supplemental Figure 1A**) for ADP-Glc PPase accompanied amounts of starch accumulated, being higher at the first stage and continuously decreasing along with development. A remarkable difference compared to wheat grains is that in castor bean, the ADP-Glc PPase exhibited no phosphorylation at any DPP stage of seeds (**Supplemental Figure 1B**).

It is worth noting that our results reveal the proteolytic degradation of control actin at 17–27 DPA in wheat (**Figure 1C**) and 35-50 DPP in castor bean (**Supplemental Figure 1C**). This is in agreement with reports indicating that at the advanced stages of development the seed may contain high levels of proteolytic enzymes, which with the accumulation of storage products could constitute a detriment for the stability of proteins (Ahn and Chen, 2007; Nadaud et al., 2010; Nogueira et al., 2012; Piattoni et al., 2017). The important issue concerning ADP-Glc PPase is that: (i) in the case of castor bean the absence of phosphorylation is apparent even when no proteolytic activity is observed (5–35 DPP) (**Supplemental Figure 1**); and (ii) still, its presence (phosphorylated) in wheat was observed even when there were signs of degradation (17–27 DPA) (**Figure 1**). These results support that the phosphorylation of ADP-Glc PPase would be of relevance for starch synthesis in tissues where the polysaccharide accumulation is the long-term metabolic goal for carbon partitioning. Besides, the metabolic picture is markedly different in oleaginous seeds, this fact linked with the storage product in these plants.

**Figure 2:**
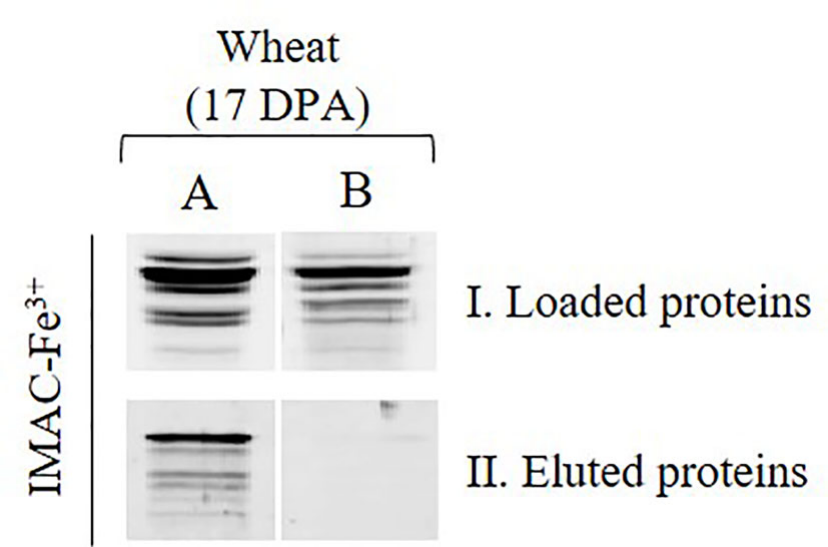
Immunodetections of ADP-Glc PPase in total wheat seed protein extract at 17 DPA **(A)** non-treated, **(B)** treated with alkaline phosphatase, (I) sample loaded on IMAC-Fe^3+^ and (II) sample eluted upon IMAC-Fe^3+^ column.

It was relevant to corroborate that ADP-Glc PPase recovery after IMAC-Fe^3+^ chromatography of the total protein extract from wheat grains was a consequence of enzyme phosphorylation, rather than an unspecific interaction of its non-phosphorylated form. For this, we performed a de-phosphorylation treatment of the whole protein extract obtained from wheat seeds at 17 DPA with alkaline phosphatase (**Figure 2**). **Figure 2A** shows that the ADP-Glc PPase from the untreated protein extract loaded for IMAC-Fe^3+^ interacted with the matrix, because of its immunodetection after elution. Conversely, for extracts incubated with alkaline phosphatase, no immunoreactive protein bands were observed after elution from the affinity chromatography (**Figure 2B**). These results confirmed that the phosphoprotein enrichment is specifically related to the presence of a post-translationally modified ADP-Glc PPase in the wheat seeds.

### Ca^2+^-Dependent Protein Kinases Are Involved in Phosphorylation of ADP-Glc PPase in Wheat Seeds

To advance in the characterization of the modification by phosphorylation of ADP-Glc PPase from wheat endosperm by protein kinases at the molecular level, we performed *in vitro* studies using enzymes produced recombinantly with high purity degree. The experimental approach considered that plant protein kinases can be classified based on structure-to-function relationships. Purposely, the classification establishes families relating protein sequence and specificity for substrates, cofactors (e.g., Ca^2+^-dependence), and other reaction conditions (Hardie, 1999). Within this categorization, it is common that the properties are similar among different plant species. In this context, the plant-specific superfamily emerges by grouping protein kinases of the type SNF1-related and Ca^2+^-dependent (SnRK-CDPK) (Vlad et al., 2008).

**Figure 3:**
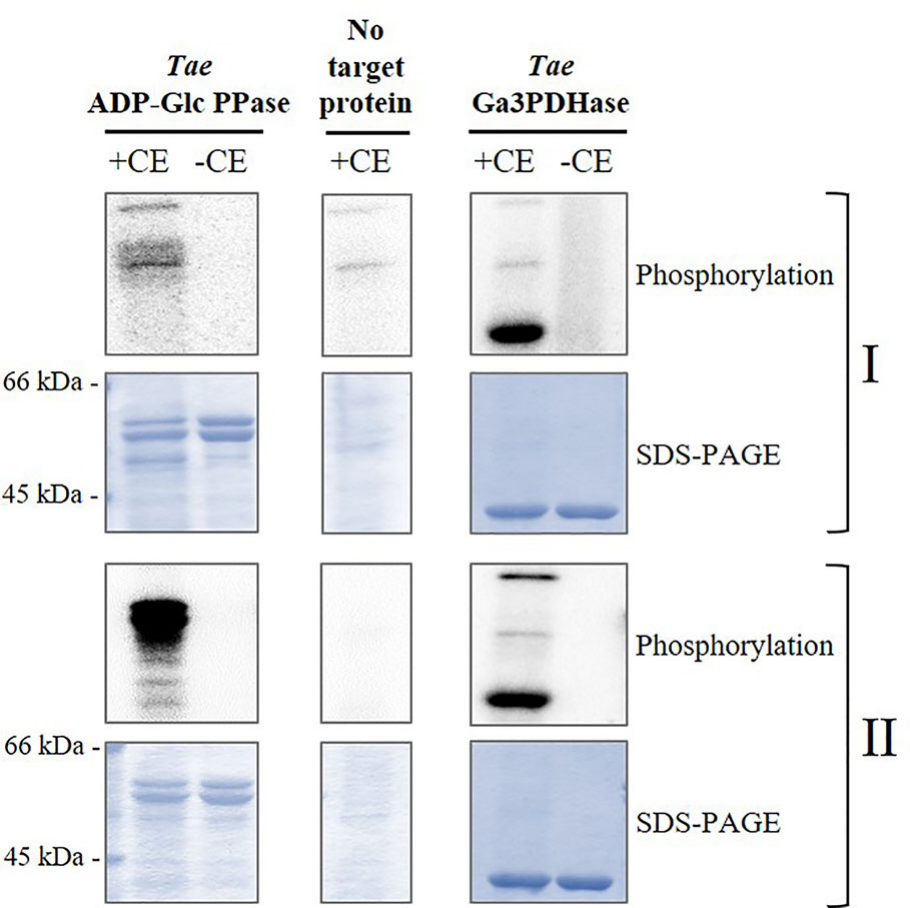
Phosphorylation of recombinant *Tae*ADP-Glc PPase by crude extracts (CE) from wheat seeds at 17 DPA. The recombinant enzyme was incubated in the presence (+) or absence (-) of total wheat seed crude extract under two different phosphorylation conditions: for (I) Ca^2+^-independent and (II) Ca^2+^-dependent protein kinases. After the phosphorylation reaction with [^32^P]ATP, the presence of radioactive label was detected by exposure of the SDS-PAGE gel to a Storage Phosphor-Screen. As controls, we used seed crude extract without further addition and the recombinant *Tae*Ga3PDHase, an enzyme already reported as a target of phosphorylation in wheat (Piattoni et al., 2017). The protein phosphorylation was performed from three independent technical replicates.

Based on the above detailed, we first incubated the recombinant heterotetrameric wheat endosperm ADP-Glc PPase (*Tae*ADP-Glc PPase) with crude extracts from wheat seeds at the 17 DPA stage of development in media containing [^32^P]ATP and optimal conditions for the activity of different plant protein kinases. These conditions allowed to assay either, Ca^2+^-independent (SnRK1) or Ca^2+^-dependent (CDPK and SOS2) protein kinases (Piattoni et al., 2011; Piattoni et al., 2017; Rojas et al., 2018). As shown in **Figure 3**, *Tae*SL was poorly phosphorylated under conditions promoting SnRK1 activity (left panels), even when this kinase was able to modify the wheat NAD^+^-dependent glyceraldehyde-3P dehydrogenase (EC 1.2.1.12, *Tae*Ga3PDHase) used as control of positive phosphorylation (right panels) (Piattoni et al., 2011). Conversely, marked phosphorylation of *Tae*ADP-Glc PPase was evident in media favoring Ca^2+^-dependent protein kinases (**Figure 3**).

**Figure 4:**
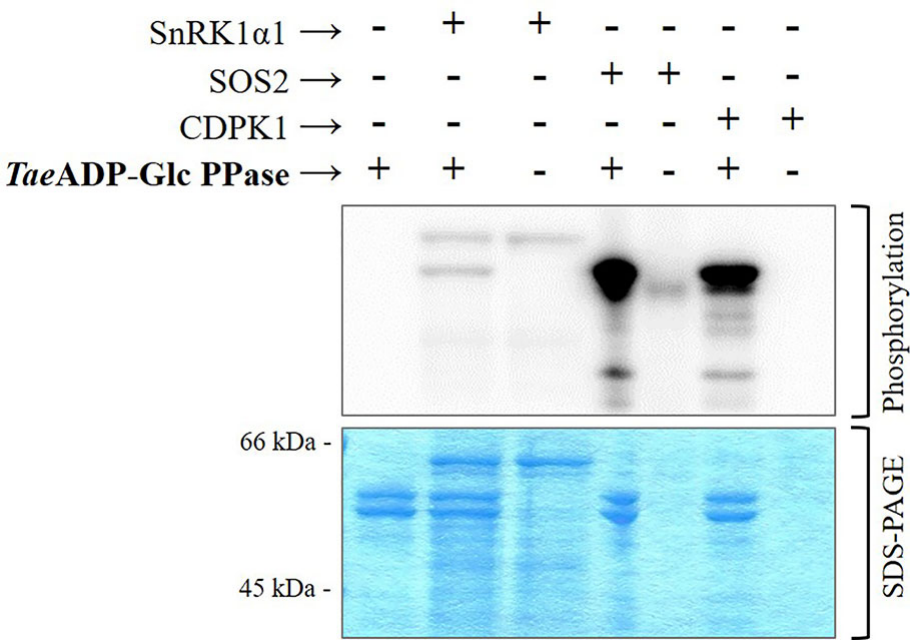
Phosphorylation of recombinant *Tae*ADP-Glc PPase by recombinant protein kinases: SnRK1α1 from *Arabidopsis thaliana*, SOS2 from *Malus domestica* and CDPK1 from *Solanum tuberosum*. After the phosphorylation reaction with [^32^P]ATP, the presence of radioactive label was detected by exposure of the SDS-PAGE gel to a Storage Phosphor-Screen. The protein phosphorylation was performed from three independent technical replicates.

The above described higher capacity of Ca^2+^-dependent protein kinases was confirmed performing *in vitro* experiments using highly purified, recombinant forms of SnRK1α1 (Ca^2+^-independent), SOS2, and CDPK1 (Ca^2+^-dependent) kinases from different plants to phosphorylate *Tae*ADP-Glc PPase. Results illustrated by **Figure 4** indicate that the incorporation of radioactive phospho-moieties from [^32^P]-ATP into the ADP-Glc PPase was significantly higher when the enzyme was incubated with SOS2 and CDPK1 in comparison with that performed with the SnRK1α1 plant protein kinase. These data support a scenario where the phosphorylation of ADP-Glc PPase by Ca^2+^-dependent protein kinases would contribute to direct metabolism toward the active synthesis of starch in wheat seeds. This fact would be relevant in a plant tissue that, at complete development, mainly drives assimilated carbon to produce the polysaccharide for a long-term reserve component.

### The L Subunit of ADP-Glc PPase Is Majorly Phosphorylated in Wheat Seeds

**Figure 5:**
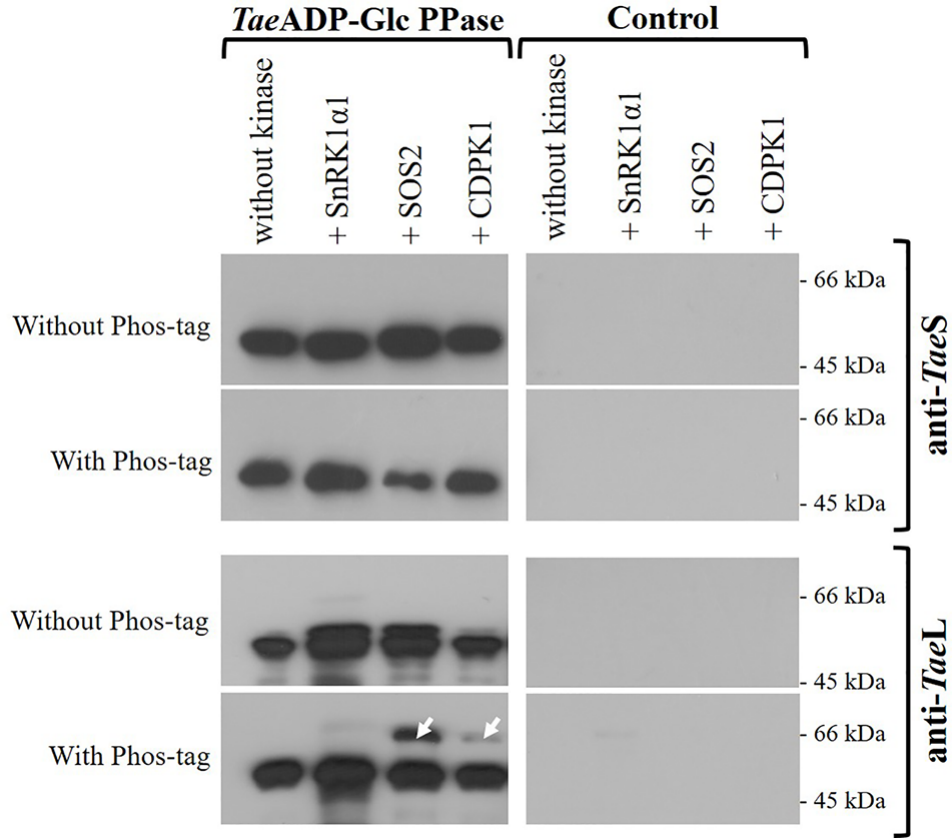
Immunodetection of S and L subunits of phosphorylated recombinant *Tae*ADP-Glc PPase. The heterotetrameric enzyme was phosphorylated with the respective recombinant protein kinase and then resolved by SDS-PAGE with or without Phos-tag, with subsequent electrotransfer and immunodetection using specific antibodies anti-*Tae*L or anti-*Tae*S. Lanes without *Tae*ADP-Glc PPase are shown as control. The white arrows indicate the phosphorylated delayed peptides. The protein phosphorylation was performed from three independent technical replicates.

Since the functional ADP-Glc PPase found in the wheat endosperm is a heterotetrameric enzyme, we sought to determine if its phosphorylation involves the indistinct modification of both subunits. For such a purpose, we performed the incubation of *Tae*ADP-Glc PPase with SnRK1α1, SOS2, or CDPK1 under conditions of phosphorylation using non-radioactive ATP. Afterward, the samples were analyzed by SDS-PAGE (either alone or containing Phos-tag, to explore the lower mobility of phosphorylated proteins) followed by electro-transference and detection using antibodies specific for each ADP-Glc PPase S or L subunits. **Figure 5** shows that after treatment assuring modification by the Ca^2+^-dependent protein kinases, the delay in migration was only evident for the L polypeptide, suggesting that this is the subunit that is the target of phosphorylation.

Further experiments gave support to the specificity of the protein kinases to modify the L subunit of *Tae*ADP-Glc PPase. Indeed, the incubation of the purified recombinant *Tae*S or *Tae*L forms with crude extracts from wheat seeds and [^32^P]ATP rendered SDS-PAGE profiles revealing phosphorylation of both subunits but at a different degree. As illustrated by **Supplemental Figure 3**, significantly higher incorporation of radioactivity took place on the *Tae*L protein. Also, in the analysis of the samples from the incubation of *Tae*S or *Tae*L subunits with the recombinant plant protein kinases using the approach of western-blots from SDS-PAGE with Phos-tag, the lower mobility was only observed for L polypeptide (**Supplemental Figure 4**). These results strongly agree with the key (tissue-specific) regulatory role assigned to the L subunit of heterotetrameric ADP-Glc PPases (Crevillén et al., 2003; Ballicora et al., 2004; Ventriglia et al., 2008; Ferrero et al., 2018; Figueroa et al., 2018), suggesting that its post-translational modification by phosphorylation would be relevant for starch synthesis in wheat seeds.

## Conclusions

In this work, we report that wheat seed ADP-Glc PPase undergoes progressive phosphorylation along with grain development. The modification of the enzyme increased, as well as the specific activity, during the transition and the beginning of the maturation steps of development. These changes followed the pattern of active synthesis of starch, which is the principal carbon reserve in this grain. The plant protein kinases involved in the phosphorylation of *Tae*ADP-Glc PPase are of the type Ca^2+^-dependent, and studies with recombinant enzymes support the specific action of SOS2 and CDPK1 kinases. Notably, the post-translational modification of ADP-Glc PPase was absent in seeds of castor bean, which accumulate lipids instead of carbohydrates as a reserve. Results reported herein suggest that phosphorylation of ADP-Glc PPase in seeds of grasses would be relevant for the synthesis of starch. Future studies will shed light on the actual effects of the phosphorylation on the stability or allosteric regulation (or both) of the wheat endosperm ADP-Glc PPase. A critical role of phosphorylation on the modulation of this enzyme is in good agreement with the limiting role it plays for the production of the main reserve polysaccharide in bacteria and plants (Ballicora et al., 2003; Ballicora et al., 2004). The characterization of the post-translational modification at a molecular level indicated that in the heterotetrameric *Tae*ADP-Glc PPase, the L subunit is at least the major target of phosphorylation. In other plant species, the L subunit also has a primary function in tissue-specific regulation of the catalytic activity exerted by the S subunit (Crevillén et al., 2003; Ballicora et al., 2004; Ballicora et al., 2005; Ventriglia et al., 2008; Ferrero et al., 2018; Figueroa et al., 2018).

This work provides evidence on the post-translational phosphorylation of ADP-Glc PPase in wheat seeds, thus complementing previous predictive phosphoproteomic studies (Lohrig et al., 2009; Kötting et al., 2010; Ma et al., 2014). This modification of the enzyme limiting the pathway of starch biosynthesis would be part of a proposed general mechanism in which protein kinases critically phosphorylate different enzymes involved in the metabolism of the polysaccharide in plants (Kötting et al., 2010; Geigenberger, 2011; Goren et al., 2018). In this framework, phosphorylation would chiefly orchestrate the functioning of the starch metabolism in combination with redox modification and the formation of complexes between different proteins (Lohrig et al., 2009; Kötting et al., 2010; Geigenberger, 2011; Ma et al., 2014; Tetlow et al., 2015; Goren et al., 2018). These combined processes would operate as an effective mechanism to optimize the accumulation of the polysaccharide in seeds of wheat and other grass crops. Concerning post-translational modification, the wheat endosperm ADP-Glc PPase was distinctively characterized as insensitive to the redox regulation demonstrated for the enzyme from other plant sources (Ballicora et al., 2004; Linebarger et al., 2005; Tuncel et al., 2014; Ferrero et al., 2018). Thus, results reported herein provide new information about the mechanism involved in starch synthesis in grasses.

The gained information reported at present opens many research approaches in the way to reach a better understanding (at the molecular level) of starch synthesis in wheat (and other cereals producing the polysaccharide as a prime component). A critical issue to investigate is related to protein chemistry studies (including mass spectrometry) to reach the identification of the specific residues in the enzyme that are the target of protein kinases. This latter characterization could be followed by the production of phosphomimetic forms of the *Tae*ADP-Glc PPase, which would allow analyzing how phosphorylation modifies its kinetic, regulatory, and stability properties. In this scenario, the possibility that phosphorylated *Tae*ADP-Glc PPase would better interact with other enzymes and proteins is an issue needing attention in future research. Another topic to be explored refers to gaining detail of the specific Ca^2+-^dependent protein kinase from wheat involved in the post-translational modification. This subject is of complex understanding, considering that *T. aestivum* is a hexaploid organism, a product of broad hybrid mix in the breeding process (Appels et al., 2018). At present, genomic information on *Triticum aestivum* L. identified fifteen and twenty genes coding for SnRK1 (Perochon et al., 2019) and CDPK (Li et al., 2008), respectively.

Future studies will be central for the design of strategies and biotechnological tools to improve yields in agriculture. Starch, as the primary feedstock of many crops, is currently the leading world supplier for caloric demands of animals (including humans). Besides, starch is a natural product of high relevance for the development of biofuels and bioplastics, which are critical products to cope with the world-wide challenges tied to demographic expansion and climate change.

## Data Availability Statement

All datasets generated for this study are included in the article/**Supplementary Material**.

## Author Contributions

DF, CP, MA, BR, MH, MB, and AI conceived and designed the experiments. AI wrote the paper. DF, BR, and MH performed the experiments. DF, CP, MA, MB, and AI analyzed the data. AI, MA and MB contributed reagents, materials, and analysis tools.

## Funding

This work was supported by the National Science Foundation (grant MCB 1616851 to MAB), and by grants from ANPCyT (PICT 2017 1515 and PICT 2018 00929 and to AAI; PICT 2015 0634 to MDAD), UNL (CAID 2016, PIC 50420150100053LI, to AAI) and CONICET (PUE 2016 0040 to IAL). MDAD and AAI are members of the Research Career from CONICET. DF and BR are doctoral fellows from CONICET.

## Conflict of Interest

The authors declare that the research was conducted in the absence of any commercial or financial relationships that could be construed as a potential conflict of interest.

## Supplementary Material

Supplemental Table 1 Determinations of weight, starch, and TAG contents, ADP-Glc PPase activity and total soluble proteins in castor bean at different development stages. DPP, days post-pollination. The determinations are the mean of at least three independent set of data that were reproducible within ±10%.

Supplemental Figure 1 Immunodetections in castor bean samples throughout development: **(A)** total proteins and **(B)** phosphoproteins purified by IMAC-Fe^3+^ evaluated with anti-ADP-Glc PPase obtained from the purified enzyme of leaves of *Spinacia oleracea* (Gómez-Casati and Iglesias, 2002) and **(C)** control of actin in total proteins. Crude extracts preparation and phosphoprotein purification were performed from three independent biological replicates. Protein profiles of the samples are shown in Supplemental Figure 3.

Supplemental Figure 2 SDS-PAGE of wheat and castor bean seed samples throughout development: (A) total proteins (same amount in all cases) and (B) phosphoproteins purified by IMAC-Fe^3+^ (same volume of eluted protein of IMAC-Fe^3+^ in all cases).

Supplemental Figure 3 Phosphorylation of *Tae*S and *Tae*L recombinant versions by crude extracts (CE) from wheat seeds at 17 DPA. The recombinant enzyme was incubated in the presence (+) or absence (-) of total wheat seed crude extract under two different phosphorylation conditions: for (I) Ca^2+^-independent and (II) Ca^2+^-dependent protein kinases. After the phosphorylation reaction with [^32^P]ATP, the presence of radioactive label was detected by exposure of the SDS-PAGE gel to a Storage Phosphor-Screen. The protein phosphorylation was performed from three independent technical replicates

Supplemental Figure 4 Immunodetection of phosphorylated *Tae*L and *Tae*S subunits. The subunits were phosphorylated with the respective recombinant protein kinase and then resolved by SDS-PAGE with or without Phos-tag, with subsequent electrotransfer and immunodetection using specific antibodies anti-*Tae*L or anti-*Tae*S. Lanes without recombinant subunit are shown as control. The white arrows indicate the phosphorylated delayed peptides. The protein phosphorylation was performed from three independent technical replicates.
