# Supplementary material for: Phosphorylation of ADP-Glucose Pyrophosphorylase During Wheat Seeds Development
Source: Front Plant Sci. 2020 Jul 10;11:1058. doi: 10.3389/fpls.2020.01058 (PMC7366821; doi:10.3389/fpls.2020.01058)
Supplement: Supplemental Table 1 — Determinations of weight, starch, and TAG contents, ADP-Glc PPase activity and total soluble proteins in castor bean at different development stages. DPP, days post-pollination. The determinations are the mean of at least three independent set of data that were reproducible within ±10%. [file Table_1.doc]

| **Determinations** | **CASTOR BEAN (DPP)** | | | | | |
| --- | --- | --- | --- | --- | --- | --- |
|  | **5** | **10** | **20** | **25** | **35** | **50** |
| **Seed weight (mg/seed)** | 31 | 237 | 280 | 267 | 252 | 177 |
| **Starch (mg/seed)** | 0.04 | 0.55 | 0.81 | 1.01 | 0.37 | 0.06 |
| **TAG (mg/seed)** | 0.03 | 0.04 | 0.18 | 1.39 | 29.73 | 55.49 |
| **ADP‑Glc PPase activity**  **(U/mg)** | 0.209 | 0.236 | 0.272 | 0.071 | 0.041 | 0.026 |
| **Total soluble proteins (mg/seed)** | 0.073 | 0.748 | 1.285 | 2.398 | 3.498 | 1.374 |
| **Events** | *Cell*  *proliferation* | | | *Transition* | *Storage/*  *Maturation* | |
