# Supplementary figures and images for: Phosphorylation of ADP-Glucose Pyrophosphorylase During Wheat Seeds Development
Source: Front Plant Sci. 2020 Jul 10;11:1058. doi: 10.3389/fpls.2020.01058 (PMC7366821; doi:10.3389/fpls.2020.01058)

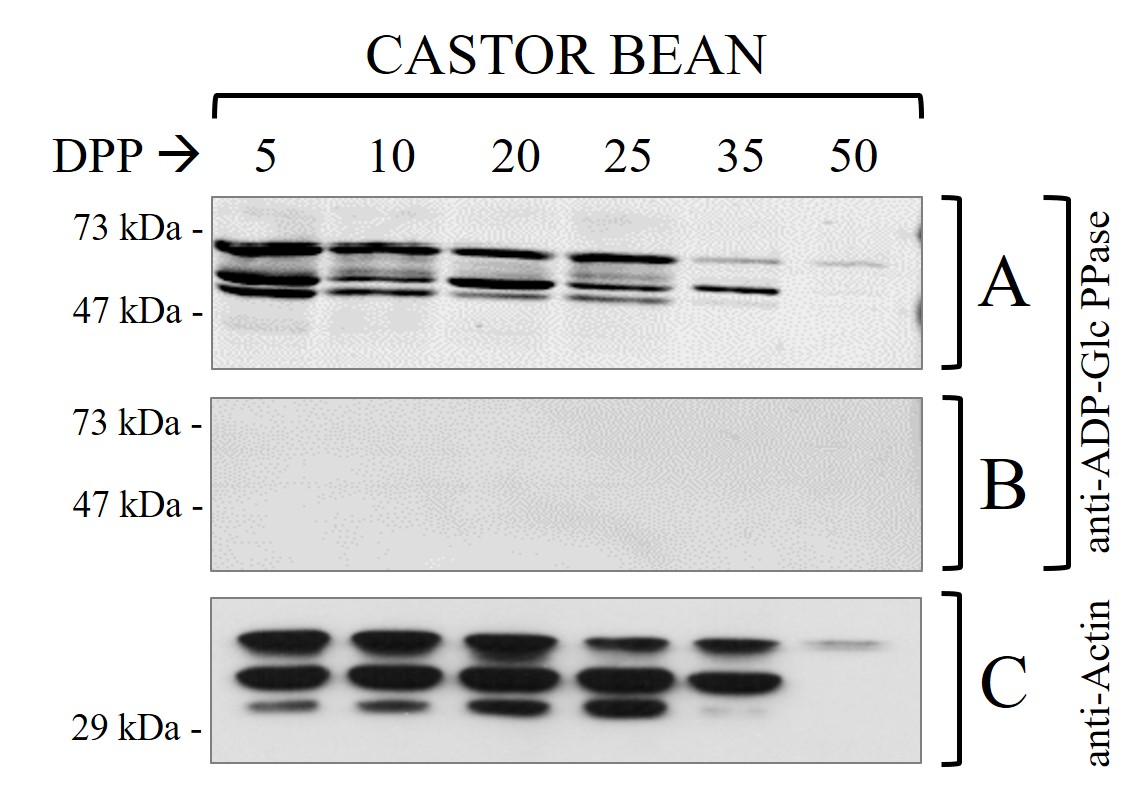

Supplement: Supplemental Figure 1 — Immunodetections in castor bean samples throughout development: (A) total proteins and (B) phosphoproteins purified by IMAC-Fe3+ evaluated with anti-ADP-Glc PPase obtained from the purified enzyme of leaves of Spinacia oleracea (Gómez-Casati and Iglesias, 2002) and (C) control of actin in total proteins. Crude extracts preparation and phosphoprotein purification were performed from three independent biological replicates. Protein profiles of the samples are shown in Supplemental Figure 3. [file Image_1.jpeg]

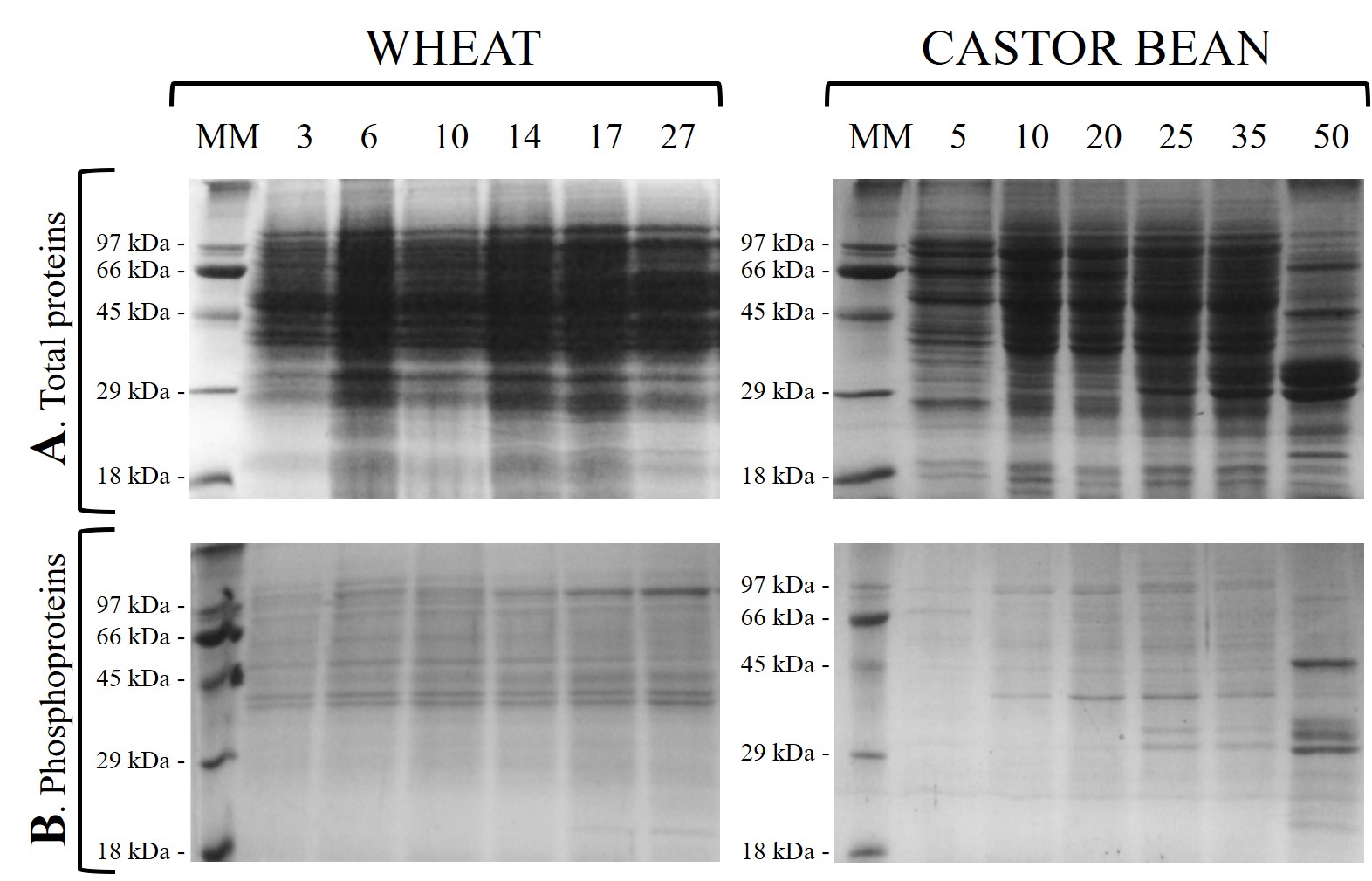

Supplement: Supplemental Figure 2 — SDS-PAGE of wheat and castor bean seed samples throughout development: (A) total proteins (same amount in all cases) and (B) phosphoproteins purified by IMAC-Fe3+ (same volume of eluted protein of IMAC-Fe3+ in all cases). [file Image_2.jpeg]

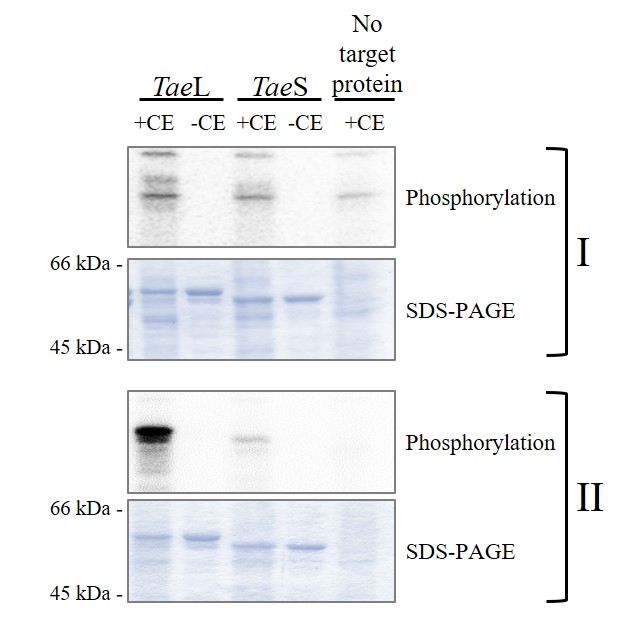

Supplement: Supplemental Figure 3 — Phosphorylation of TaeS and TaeL recombinant versions by crude extracts (CE) from wheat seeds at 17 DPA. The recombinant enzyme was incubated in the presence (+) or absence (-) of total wheat seed crude extract under two different phosphorylation conditions: for (I) Ca2+-independent and (II) Ca2+-dependent protein kinases. After the phosphorylation reaction with [32P]ATP, the presence of radioactive label was detected by exposure of the SDS-PAGE gel to a Storage Phosphor-Screen. The protein phosphorylation was performed from three independent technical replicates [file Image_3.jpeg]

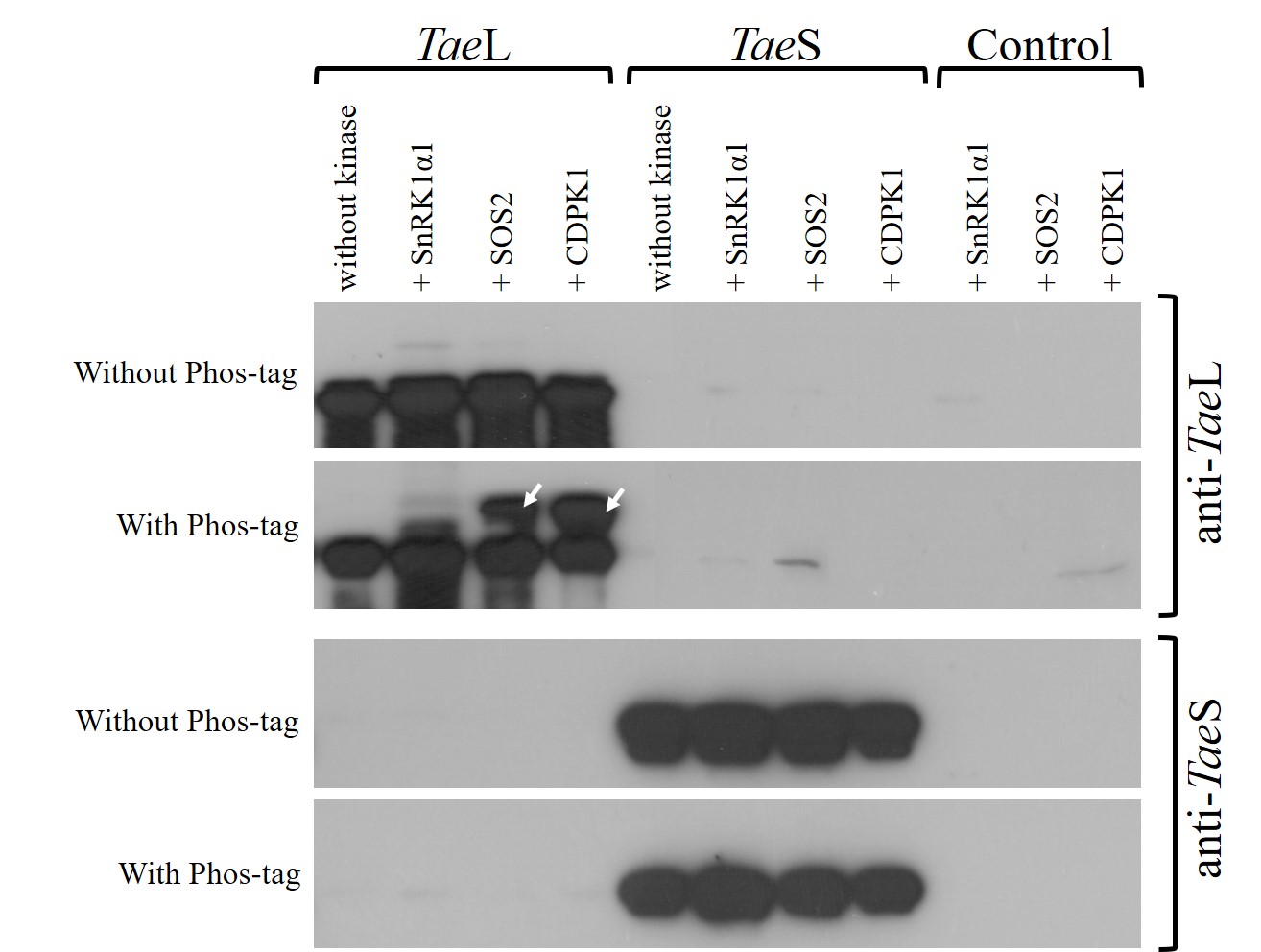

Supplement: Supplemental Figure 4 — Immunodetection of phosphorylated TaeL and TaeS subunits. The subunits were phosphorylated with the respective recombinant protein kinase and then resolved by SDS-PAGE with or without Phos-tag, with subsequent electrotransfer and immunodetection using specific antibodies anti-TaeL or anti-TaeS. Lanes without recombinant subunit are shown as control. The white arrows indicate the phosphorylated delayed peptides. The protein phosphorylation was performed from three independent technical replicates. [file Image_4.jpeg]
